# *Scheffersomyces stipitis*: a comparative systems biology study with the Crabtree positive yeast *Saccharomyces cerevisiae*

**DOI:** 10.1186/1475-2859-11-136

**Published:** 2012-10-09

**Authors:** Marta Papini, Intawat Nookaew, Mathias Uhlén, Jens Nielsen

**Affiliations:** 1 Novo Nordisk Foundation Center for Biosustainability, Department of Chemical and Biological Engineering, Chalmers University of Technology, Gothenburg, SE, 412 96, Sweden; 2 Novo Nordisk Foundation Center for Biosustainability, Technical University of Denmark, Hørsholm, DK, 2970, Denmark; 3 Novo Nordisk Foundation Center for Biosustainability, Department of Biotechnology, Royal Institute of Technology, Stockholm, Sweden

## Abstract

**Background:**

*Scheffersomyces stipitis* is a Crabtree negative yeast, commonly known for its capacity to ferment pentose sugars. Differently from Crabtree positive yeasts such as *Saccharomyces cerevisiae*, the onset of fermentation in *S. stipitis* is not dependent on the sugar concentration, but is regulated by a decrease in oxygen levels. Even though *S. stipitis* has been extensively studied due to its potential application in pentoses fermentation, a limited amount of information is available about its metabolism during aerobic growth on glucose. Here, we provide a systems biology based comparison between the two yeasts, uncovering the metabolism of *S. stipitis* during aerobic growth on glucose under batch and chemostat cultivations.

**Results:**

Starting from the analysis of physiological data, we confirmed through ^13^C-based flux analysis the fully respiratory metabolism of *S. stipitis* when growing both under glucose limited or glucose excess conditions. The patterns observed showed similarity to the fully respiratory metabolism observed for *S. cerevisiae* under chemostat cultivations however, intracellular metabolome analysis uncovered the presence of several differences in metabolite patterns. To describe gene expression levels under the two conditions, we performed RNA sequencing and the results were used to quantify transcript abundances of genes from the central carbon metabolism and compared with those obtained with *S. cerevisiae*. Interestingly, genes involved in central pathways showed different patterns of expression, suggesting different regulatory networks between the two yeasts. Efforts were focused on identifying shared and unique families of transcription factors between the two yeasts through *in silico* transcription factors analysis, suggesting a different regulation of glycolytic and glucoenogenic pathways.

**Conclusions:**

The work presented addresses the impact of high-throughput methods in describing and comparing the physiology of Crabtree positive and Crabtree negative yeasts. Based on physiological data and flux analysis we identified the presence of one metabolic condition for *S. stipitis* under aerobic batch and chemostat cultivations, which shows similarities to the oxidative metabolism observed for *S. cerevisiae* under chemostat cultivations. Through metabolome analysis and genome-wide transcriptomic analysis several differences were identified. Interestingly, *in silico* analysis of transciption factors was useful to address a different regulation of mRNAs of genes involved in the central carbon metabolism. To our knowledge, this is the first time that the metabolism of *S. stiptis* is investigated in details and is compared to *S. cerevisiae*. Our study provides useful results and allows for the possibility to incorporate these data into recently developed genome-scaled metabolic, thus contributing to improve future industrial applications of *S. stipitis* as cell factory.

## Background

The yeast *Scheffersomyces stipitis*, commonly known as *Pichia stipitis*, is a Crabtree negative, homothallic yeast, found mainly in haploid form. *S. stipis* has greater respiratory capacity than *S. cerevisiae* due to the presence of an alternative respiration system donating electrons directly to O_2_ from ubiquinone, branching out before the cytochrome C complex [1,2] and to the presence of Complex I, also lacking in *S. cerevisiae*. This Crabtree negative yeast is well known for its ability to ferment pentose sugars to ethanol, having one of the highest native capacity for xylose fermentation with yields on substrate between 0.35/ 0.44 g g^-1^[3] at low oxygen transfer rate [4]. *S. stipitis* has therefore been studied and exploited as a source of genes for the engineering of xylose metabolisms in other microorganisms and it has also been considered as a platform cell factory for production of fuels and chemicals from lignocellulose. Differently from the Crabtree positive yeast *S. cerevisiae*, the regulation of fermentation in *S. stipitis* depends on the oxygen levels, where ethanol production sets in only when oxygen becomes limiting. There are two genes responsible for ethanol production in *S. stipitis*: *ADH1* and *ADH2*, encoding the alcohol dehydrogenase complex (ADH) [5]. The activity of ADH is induced by a reduction in the oxygen tension and this regulation may be mediated by heme levels [6,7]. Under strictly anaerobic conditions, almost no ethanol is produced and the strain cannot survive longer than 1 generation. The same pattern of induction is reported for the genes of the pyruvate decarboxylase complex (PDC) and aldehyde dehydrogenase (AlDH) [8]. This behavior is profoundly different from *S. cerevisiae* where ethanol production takes place under glucose excess conditions, regardless of the availability of oxygen [9].

In light of its attractive feature to ferment pentoses, most of the work available on *S. stipitis* has been performed using xylose as the sole carbon source or in mix with other sugars and no detailed physiological studies of this organisms growing on glucose are available. Such data will be important in terms of further exploiting *S. stipitis* as a platform cell factory for production of fuels and chemicals, particularly as glucose is a dominant sugar in biomass.

Recently a genome-scale metabolic network was reconstructed for *S. stipitis*[10,11] providing an increased insight into the metabolism of this yeast. Nevertheless, still little is known about regulatory pathways in *S. stipitis*. Some regulatory proteins such as SNF1, GNC1 and HAP5 are known to share similarity to *S. cerevisiae*[12], but regulatory mechanisms have not been elucidated. Array-based expression studies during aerobic or oxygen limited conditions on glucose or xylose as carbon sources have been performed, showing that about half of the transcripts does not change significantly under the different conditions [12].

In this study, we sought to provide an insight into the metabolism of *S. stipits* during aerobic growth on glucose and to compare its patterns to the Crabtree positive yeast *S. cerevisiae* under batch and chemostat cultivations, using a systems biology approach. Besides measurement of traditional physiological parameters, we analyzed the flux distribution, intracellular metabolites levels and provide RNA-seq data to analyze gene expression levels. Additionally, to highlight the differences in regulatory network between the two yeasts, we performed *in-silico* analysis of known transcription factors. This work represents an attempt to integrate data from different systems biology tools to gain insight into the metabolism of *S. stipitis* during growth on glucose.

## Results and discussion

### Physiology of S. stipitis during aerobic growth on glucose

*S. stipitis* and *S. cerevisiae* were grown on glucose as the sole carbon source under both chemostat and batch conditions. For *S. cerevisiae*, a Crabtree positive yeast, there is a remarkable metabolic difference during these growth conditions; *S. cerevisiae* shows respiro-fermentative metabolism in the batch cultures, when glucose is available in excess, while a purely respiratory metabolism in the chemostat cultures when glucose is limiting, is observed. We were interested to see how *S. stipitis* respond to these differences in glucose concentration, and to establish eventual differences between the two different yeasts.

**Figure 1:**
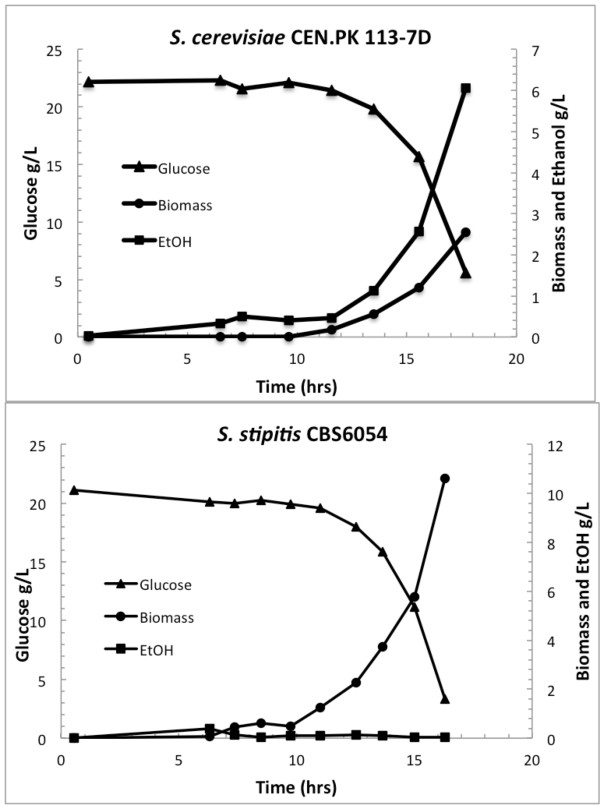
**Growth curves of *S. stipitis *and *S. cerevisiae; *aerobic, batch cultivations on minimal media with 20 g L**^**-1**^**glucose as carbon source.**

**Figure 2:**
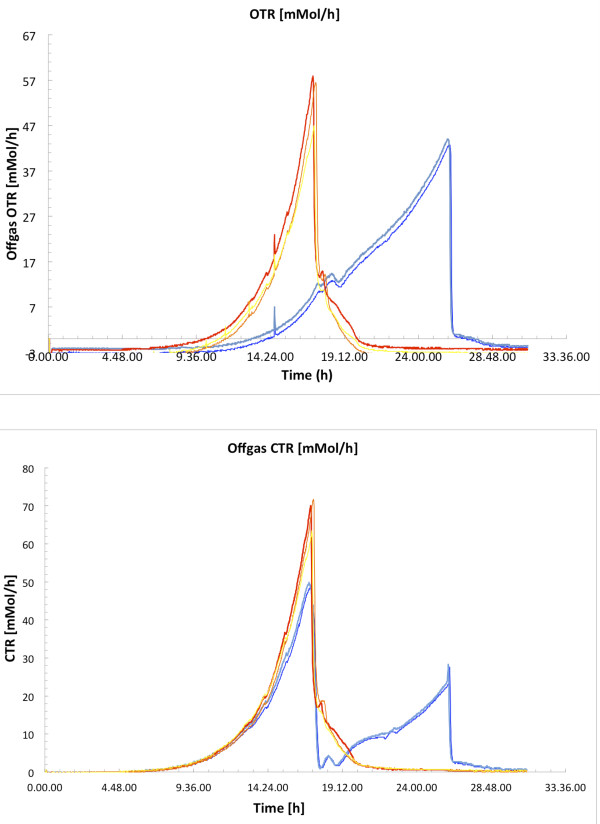
**Oxygen Transfer Rate (OTR) and Carbon Transfer Rate (CTR) in mmol/h; *S. stipitis *(red and yellow) and *S. cerevisiae *(dark and light blue) during aerobic, batch cultivations on minimal media with 20 gL**^**-1**^**glucose as carbon source.**

The growth curve of the two strains during batch cultures is shown in Figure 1. Under this condition, *S. stipitis* shows a specific growth rate of 0.47 hr^-1^ and a specific glucose consumption rate of 26.7 C-mmol (g Dry Weight)^-1^ hr^-1^, whereas *S. cerevisiae* shows a specific growth rate of 0.40 hr^-1^ and a specific glucose consumption rate of 84.5 C-mmol (g DW)^-1^ hr^-1^. For *S. stipitis*, which shows respiratory metabolism in the batch culture, no extracellular metabolites were secreted and the main products were biomass and CO_2_ with the yields on substrate listed in Table 1, showing a biomass yield of 0.55 g g (glucose)^-1^. For *S. cerevisiae*, which shows respiro-fermentative metabolism in the batch culture, ethanol and smaller amounts of glycerol, pyruvate and acetate were formed as by-products; as a consequence, the biomass yield during the glucose growth phase is much lower as shown in Table 1. In agreement with previous results, *S. stipitis* shows a high dependency on oxygen, having an oxygen transfer rate (OTR) of 0.12 g (g cell)^-1^ hr^-1^; the OTR and carbon transfer rate (CTR) of *S. stipitis* are reported in Figure 2, showing that *S. stipitis* consumes higher amount of oxygen compared with *S. cerevisiae*. The physiological patterns observed suggest that under aerobic batch cultivations *S. stipitis* shows fully respiratory metabolism. This is also confirmed by the Respiratory Quotient (RQ) calculated to be below 1 (data not shown) during the exponential growth phase.

**Table 1:** **Physiological parameters of *S. stipitis *and *S. cerevisiae*; aerobic, batch cultivations on minimal media with 20 gL**^**-1**^**glucose as carbon source**

	***S. cerevisiae***	***S. stipitis***
μ***max [h***^***-1***^***]***	0.40	0.47
***Glucose consumption rate***		
***[C-mmol /g DW/ h]***	84.5	26.7
**Ysx [g /g]**	0.17	0.55
**YsEtOH [g /g]**	0.33	0.003
**YsPyr [g /g]**	0.003	0.004
**YsAc [g /g]**	0.01	0.001

To investigate the response of *S. stipitis* to the presence of glucose excess and identify possible differences between growth in batch and chemostat, we performed chemostat cultivations at a dilution rate of 0.1 hr^-1^. Physiological analysis under this condition allowed us to establish that the yields on substrate of *S. stipitis* do not differ from those reported during batch cultivations. We could therefore conclude that *S. stipitis* shows the same behavior under the two cultivation modes and that this pattern is indeed similar to the respiratory mode observed during purely respiratory growth of *S. cerevisiae*.

### Comparison of metabolic flux distribution during aerobic batch and chemostat cultures of *S. stipitis* and *S. cerevisiae*

To address at a metabolic level the gross physiological patterns presented above, we quantified the intracellular flux distributions of *S. stipitis* and compared them to that of *S. cerevisiae* by cultivating the two yeasts on ^13^C labeled glucose under aerobic batch and chemostat conditions.

Metabolic network analysis of *S. stipitis* has been previously reported using a different method for flux resolution [13], however the overall distribution of fluxes in the metabolic network was not provided.

**Figure 3:**
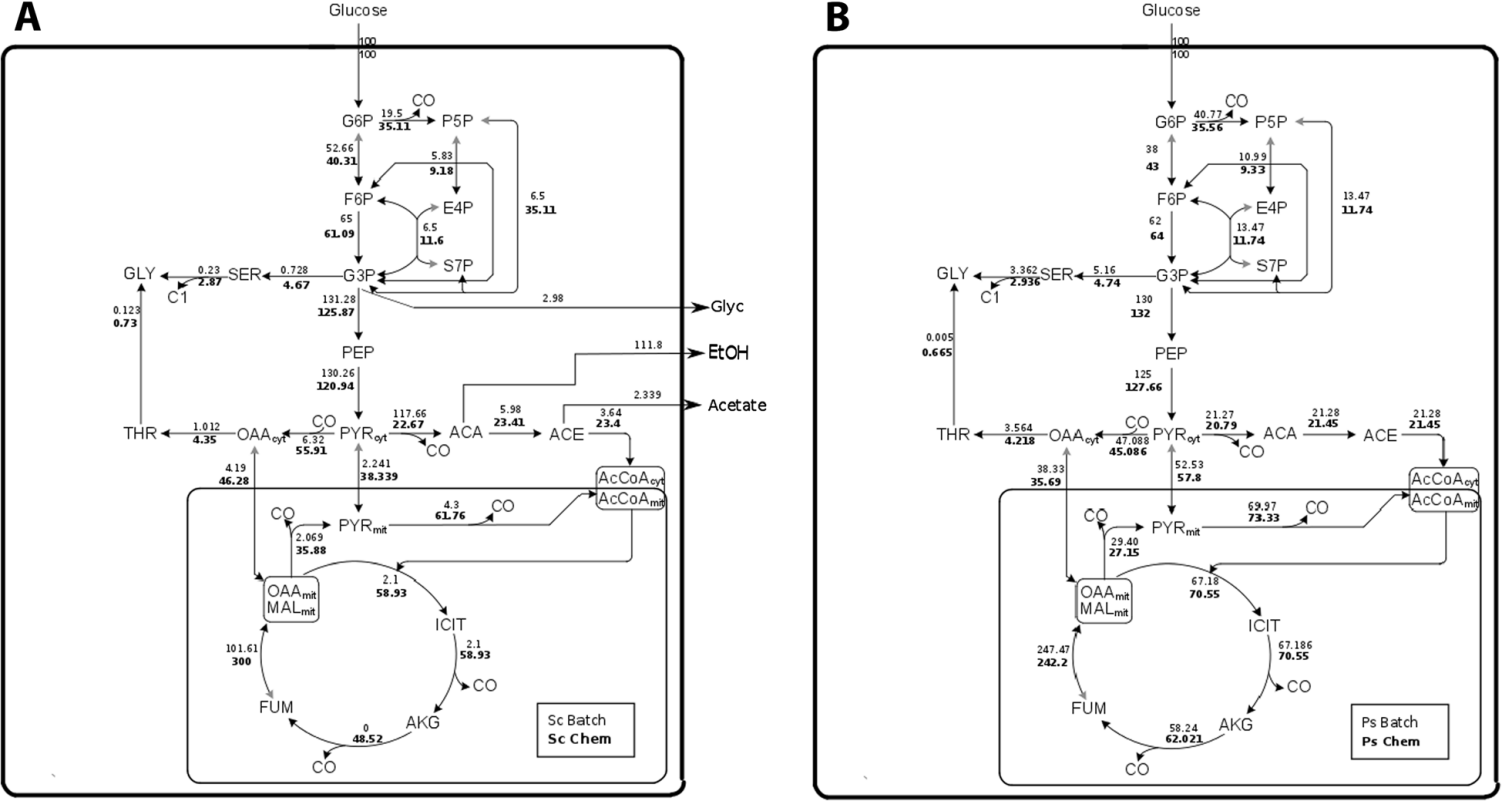
**A and B: Intracellular flux distribution of *S. cerevisiae *(A) and *S. stipitis *(B); Flux distribution was resolved during batch on glucose 5 gL**^**-1**^**as carbon source (normal font) and during chemostat (bold font) cultivations on glucose 2 gL**^**-1**^**at dilution rate 0.1 hr**^**-1** ^***S. cerevisiae *= Sc; *S. stipitis *= Ps.**

Here, for the first time, we provide a distribution of the intracellular metabolic network of *S. stipitis*; this was calculated based on the summed fractional labeling (SFL) of proteinogenic amino-acids in combination with metabolic flux analysis (MFA). The distribution of the metabolic network of *S. stipitis* under the two cultivations conditions was compared to *S. cerevisiae*, addressing relevant differences and similarities that are shown in Figure 3A (*S. stipitis*) and Figure 3B (*S. cerevisiae*). The fluxes shown are net fluxes, normalized on the glucose uptake. The flux values reported for *S. cerevisiae* are in good agreement with those reported by Gombert *et al.*[14]. Interestingly, the flux network of *S. stipitis* does not show significantly different values between batch and chemostat conditions (Figure 3A). It is further seen that the flux values found for *S. stipitis* have similarities to the flux distribution observed for *S. cerevisiae* during respiratory growth.

In *S. stipitis,* no significant differences were present in the lower glycolytic flux or in the tricarboxylic acid cycle (TCA) during batch and chemostat, however the flux towards the pentose phosphate pathway was estimated to be slightly lower during chemostat cultures. Additionally, no external flux of metabolites (metabolites secretion) was reported, in agreement with the measured physiological parameters. The flux values of the pyruvate dehydrogenase (PDH) and of the pyruvate transport into the mitochondria are comparable between the two conditions, as well as fluxes through the TCA. Our results indicate that the metabolic fluxes of *S. stipitis* under the two cultivations conditions do not show remarkable differences. In contrast, in *S. cerevisiae,* the flux distribution in batch and chemostat conditions shows extremely different patterns, reflecting the metabolic states arising as a consequence of regulatory phenomena due to glucose repression [14,15].

Remarkable differences are found in the tricarboxylic acid cycle (TCA): while in *S. cerevisiae* highly different values are found in batch and chemostat, as previously reported [14], in *S. stipitis* high values of the TCA fluxes are found regardless of the cultivation mode, however, in *S. stipitis,* the TCA fluxes during chemostat cultures are slightly higher than during batch culture. In *S. stipitis*, the flux through the oxidative part of the pentose phosphate pathway (PPP) is higher, similarly to *S. cerevisiae* during respiratory conditions. The flux distribution at the pyruvate branch point in *S. stipitis* also shows substantial differences with *S. cerevisiae* during batch cultures. First, the flux through the pyruvate decarboxylate reaction (PDC) in *S. cerevisiae* shows remarkably high values, indicating a flux towards acetaldehyde formation during glucose excess conditions; instead, during oxidative growth, this flux presents a lower value, comparable to that reported for *S. stipitis*. Additionally, during respiro-fermentative growth, *S. cerevisiae* shows secretion of intracellular metabolites (mainly ethanol but also minor amount of glycerol, pyruvate and acetate), as previously mentioned.

The flux corresponding to the anaplerotic reaction of pyruvate carboxylase (PYC) is also comparable within the two growth conditions for *S. stipis*, whereas in *S. cerevisiae* this flux is almost 10 times higher in the chemostat culture compared with the batch culture, however, when the flux between yeasts is compared, in *S. stipitis* is slightly lower than these reported during oxidative growth in *S. cerevisiae*. A similar pattern is observed for pyruvate import into the mitochondria: this flux is low during fermentative growth of *S. cerevisiae* and about 16 times higher in the chemostat, while in *S. stipitis* this flux shows constitutively high values. Other differences are found in the pyruvate dehydrogenase (PDH) flux: in *S. cerevisiae* during respiro-fermentative growth acetyl-CoA formation mainly occur through the PDH-bypass (PDC, acetaldehyde dehydrogenase and acetyl-CoA synthase), while, during oxidative growth, acetyl-CoA is mainly generated through the PDH reaction [16]; in agreement with previous studies we reported a value of 4.3 for the batch and 61.7 for the chemostat culture. In contrast, *S. stipitis* shows similar flux values at the two growth conditions and, in *S. stipitis,* the PDH flux is slightly higher than that found in *S. cerevisiae* during oxidative growth, suggesting that in *S. stipitis* mitochondrial acetyl-CoA formation mainly occurs through the pyruvate dehydrogenase reaction.

A difference between the flux distribution of *S. stipitis* and that of *S. cerevisiae* during oxidative growth is found in the malic enzyme flux (MAE); the flux reported by *S. stipitis*, comparable in the two conditions, is higher than that presented by *S. cerevisiae* in batch cultivations, but lower than that found during oxidative growth of *S. cerevisiae*[13]. This result, together with the lower flux value observed for the anaplerotic decarboxylation of pyruvate to oxaloacetate (PYC), suggests a different tuning of anaplerotic reactions in the two yeasts, being lower in *S. stipitis*, in agreement with the results reported by Fiaux *et al.*[13].

Intracellular flux distribution analysis supported the presence, in *S. stipitis,* of one main metabolic mode, showing similar patterns to those observed for *S. cerevisiae* during respiratory growth; however, minor differences between the respiratory growth of *S. stipitis* and the respiratory growth of *S. cerevisiae* were identified. In *S. cerevisiae* instead, as previously well characterized, physiology and flux network distribution differ substantially between the two conditions as a consequence of regulatory phenomena not occurring in *S. stipitis*.

### Intracellular metabolites analysis of *S. stipitis* during aerobic batch cultivations

To gain further insight into the difference observed between *S. stipitis* and *S. cerevisiae* under glucose excess conditions, we compared metabolome profiles of the two yeasts under batch cultivations. Metabolic fingerprinting allows the quantification of intracellular metabolites, providing realistic information about the metabolic state of the cell under a certain condition. After statistical analysis, it was possible to identify different representation of metabolites involved in different pathways between the two yeasts, as shown in Table 2. Tables 3, 4 and 5 report the fold-change (*S. stipitis* vs *S. cerevisiae*) of metabolites with significant p-values, listed accordingly to their super and sub-pathway. Changes in metabolites belonging to the amino acids pattern are reported in Table 3, where it is possible to observe that most amino-acids are present in slightly lower concentrations in *S. stipitis*. Adenine content, instead, is remarkably increased in *S. stipitis* (Table 3) probably as a result of a higher specific growth rate. Interestingly, the high fold-change of citramalate and phenylacetate show that these metabolites are present in high concentration in *S. stipitis*, as their presence in *S. cerevisiae* is very low. Citramalate (2-methylmalate), a metabolite involved in the leucine biosynthetic pathway, synthesized from pyruvate and acetyl-CoA, shows a high fold-change in *S. stipitis*; for this reason, we sought for the presence of a reaction generating citramalate in *S. stipitis*: despite a reaction leading to the formation of citramalate is present in the recently published genome-scale metabolic model iSS884, the gene coding for this reaction has not been identified and its activity never proved. We sought the presence of citramalate synthase by blasting the protein sequence from microorganisms having this reaction (e.g. *cimA* from *Methanocaldococcus jannaschii)* against the translated proteins from the genome sequence of *S. stipitis*. We found high similarity to 2-isopropylmalate synthase (IPMS, Gene ID: 4850963) and we thus suggested that the isopropylmalate synthase might have a role in generating citramalate.

**Table 2:** Results from statistical analysis on intracellular metabolite analysis

**Statistical Comparison**				
Welch’s Two Sample t-Test	Total number of Biochemicals with	Biochemicals	Total number of biochemical with	Biochemicals
	p ≤ 0.05		0.05 < p <0.1	
		p ≤0.05		0.05 < p < 0.1
Ss /Sc	114	**17**/**97**	16	**1**/**15**

**Table 3:** **Fold change of amino acids and nucleotides pathway with relative p-values (*Ss*** **=** ***S. stipitis*; *Sc*** **=** ***S. cerevisiae*)**

**SUPER PATHWAY**	**SUB PATHWAY**	**BIOCHEMICAL NAME**	**Protein Normalized Fold Change Ss / Sc**	**P-VALUE**
**AMINO ACIDS**	*Glycine, serine and threonine metabolism*	beta-hydroxypyruvate	0,22	0,0221
		O-acetylhomoserine	0,14	0,0068
		threonine	0,19	0,0118
	*Alanine and aspartate metabolism*	N-carbamoylaspartate	0,32	0,0450
	*Glutamate metabolism*	glutamate, gamma-methyl ester	0,14	0,0010
		glutamine	0,26	0,0291
		N-acetylglutamate	0,17	0,0156
	*Histidine metabolism*	histidine	0,22	0,0031
	*Phenylalanine & tyrosine metabolism*	phenyllactate (PLA)	0,28	0,0450
		phenylalanine	0,22	0,0180
		phenylacetate	1,67	0,0444
		tyrosine	0,39	0,0300
		N-acetylphenylalanine	0,21	0,0087
	*Valine, leucine and isoleucine metabolism*	isoleucine	0,16	0,0030
		valine	0,30	0,0134
		alpha-hydroxyisovalerate	0,25	0,0472
		citramalate	3,15	0,0311
	*Urea cycle; arginine-, proline-, metabolism*	arginine	0,12	0,0036
		proline	0,19	0,0031
		citrulline	0,12	0,0101
**NUCLEOTIDES**	*Purine metabolism, (hypo)xanthine/inosine containing*	inosine	0,15	0,0056
	*Purine metabolism, adenine containing*	adenine	38,76	0,0018

**Table 4:** **Fold change of metabolites from carbohydrates and energy metabolism pathway with relative p-values (*Ss*** **=** ***S. stipitis*; *Sc*** **=** ***S. cerevisiae*)**

**SUPER PATHWAY**	***SUB PATHWAY***	**BIOCHEMICAL NAME**	**Protein Normalized Fold Change Ss / Sc**	**P-VALUE**
**CARBOHYDRATES**	*Glycolysis, gluconeogenesis, pyruvate metabolism*	2-isopropylmalate	0,31	0,0164
		glucose	0,21	0,0238
		fructose 1,6-diphosphate, glucose 1,6-diphosphate	0,22	0,0097
		pyruvate	0,17	0,0145
		2,3-butanediol	0,06	0,0314
		trehalose	4,06	0,0174
	*Glyoxylate and dicarboxylate metabolism*	oxalate (ethanedioate)	0,18	0,0337
	*Nucleotide sugars, pentose metabolism*	arabitol	317,22	< 0, 001
		ribitol	46,97	< 0, 001
		ribulose	4,31	0,0057
		arabinose	0,28	< 0, 001
		xylulose	0,33	0,0048
**ENERGY METABOLISM**	*Krebs cycle*	citrate	0,45	0,0296
		succinate	0,56	0,0211
		fumarate	7,52	0,0127
		malate	4,75	0,0196
	*Oxidative phosphorylation*	phosphate	0,22	0,0338

**Table 5:** **Fold change of metabolites involved in lipid metabolism with relative p-values (*Ss*** **=** ***S. stipitis*; *Sc*** **=** ***S. cerevisiae*)**

**LIPIDS**	***Essential fatty acid***	**linoleate (18:2n6)**	**2,35**	**< 0, 001**
	*Long chain fatty acid*	palmitoleate (16:1n7)	0,17	0,0035
	*Fatty acid, dicarboxylate*	2-hydroxyglutarate	2,23	0,0187
	*Glycerolipid metabolism*	glycerol	0,10	0,0210
		choline	3,11	0,0319
	*Lysolipid*	2-palmitoleoylglycerophosphoethanolamine*	0,29	< 0, 001
		1-oleoylglycerophosphoethanolamine	0,45	0,0019
		2-linoleoylglycerophosphoethanolamine*	9,17	0,0387
		1-palmitoleoylglycerophosphoinositol*	0,22	0,0430
		2-oleoylglycerophosphoinositol*	0,32	0,0294
	*Sphingolipid*	sphinganine	0,26	0,0210

Table 4 shows the changes in metabolites involved carbohydrates and energy metabolism. Here, it is striking the high fold-change observed for ribulose and polyols such as arabitol and ribitol. Previous studies in *Aspergillus niger* demonstrated that polyols are synthesized under oxygen limiting conditions, acting as carbon storage compounds but also having a role in the maintenance of the osmotic and redox balance [17,18]. Based on these results, we suggested the capability of *S. stipitis* to utilize the arabinose assimilation pathway in the opposite direction, producing arabitol and ribitol via the PPP pathway [19]. The content of trehalose is also found to be significantly higher in *S. stipitis*; the increased presence of this metabolite, together with the increased adenine content compared to *S. cerevisiae*, might be linked to the increased biomass production. This observation is in agreement with previous results suggesting that Crabtree negative yeasts accumulates reserve carbohydrates upon glucose pulse [20]. Metabolites of the energy metabolism do also show differences between the two yeasts. Fumarate and malate contents are higher in *S. stipitis* whereas succinate and malate are present in slightly lower amount. Pyruvate and acetyl-CoA are also less abundant in *S. stipitis*, however it is unfortunately not possible to distinguish between the cytosolic and mitochondrial fraction.

Intracellular levels of lipids are shown in Table 5. The fatty acid linoleate (18:2) and the phospholipid 2-linoleoyl glycero-phosphoethanolamine have high fold-change as these lipids are naturally not abundant in *S. cerevisiae*[21]. Choline content is also found to be present in higher amount in *S. stipitis*. 2-hydroxyglutarate, originating from the TCA intermediate 2-oxoglutarate is found to be present at higher levels in *S. stipitis*[22].

Intracellular metabolome analysis highlighted several differences in metabolites patterns of carbohydrates, energy and fatty acids metabolism. Despite some of these results might be directly connected to what observed at a phenotypic level, other differences could not be captured by physiological analysis, thus proving the validity of metabolome analysis in providing useful information for metabolic characterization.

### RNA sequencing from aerobic cultivations

Not much is known about the regulatory pathways of *S. stipitis* and only a few works describing gene expression levels in *S. stipitis* have been performed. To determine gene expression levels during growth on glucose, we analyzed the transcriptome of *S. stipitis* in a high-throughput fashion, using RNA-seq to compare mRNAs extracted from aerobic batch and chemostat cultivations to those of *S. cerevisiae*[23]. Jeffries *et al.* analyzed gene expressions through NimbleGen expression arrays, comparing the transcriptional response to oxygen limitation on glucose and xylose [12,24] and showed that half of the transcript do not change expression significantly under the four different conditions. Recently Yuan *et al.* sequenced the transcriptome of *S. stipitis* growing on glucose and xylose, identifying 214 ORFs whose expression is changed during growth on the two different carbon sources [25].

**Figure 4:**
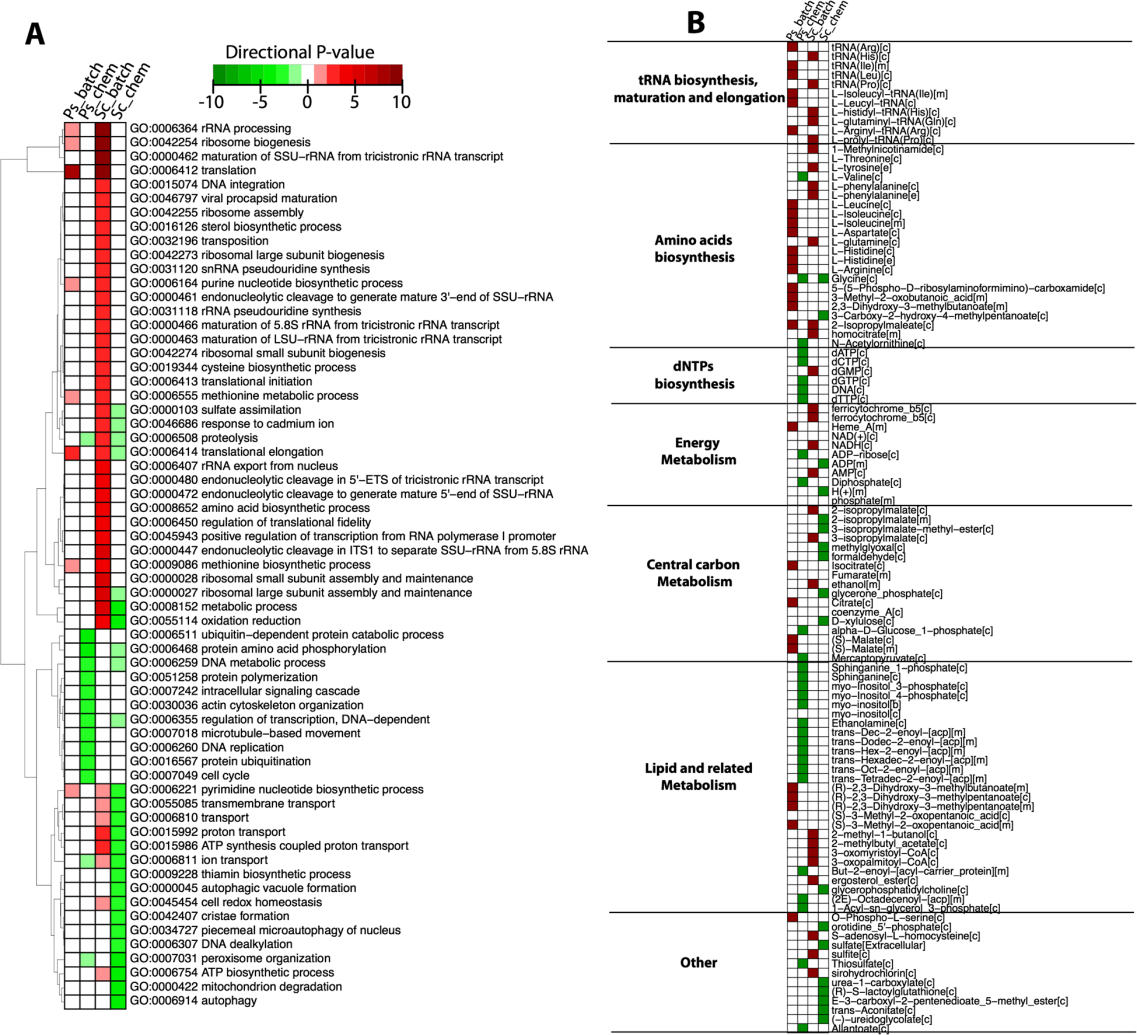
**A: Heatmap of Gene Ontology terms (GO) of *S. cerevisiae *and *S. stipitis *under batch and chemostat cultivations.B**: heatmap of Reporter Metabolites (RM), listed according to pathway they belong. Legend: Sc batch = *S. cerevisiae* batch; Sc chem = *S. cerevisiae* chemostat; Ps batch = *S. stipitis* batch; Ps chem = *S. stipitis* chemostat.

From the RNA sequencing we obtained in average of 5.95 million pair-end reads for each sample, of which > 90% mapped to *S. stipitis* CBS6045 and to *S. cerevisiae* S288c genome. We used RNA-seq data to describe the transcriptome in a comparative fashion between the two yeasts within the two conditions. To this end, we firstly used RNA-seq data in a qualitative way to identify significant Gene Ontology terms (GO, Figure 4A) and reporter metabolites (Figure 4B) over-represented in each condition and compare them between the two yeasts. In Figure 4 (A and B) the p-values are shown as directional p-values, where green indicates higher expression in chemostat (negative p-value) and red indicates higher in batch. In section A, it is possible to observe that while for *S. cerevisiae* there are different GO terms up-regulated in one or in the other condition, for *S. stipitis* only a few terms show remarkable differential expression; among these we find growth-related terms such as rRNA elongation, ribosome biogenesis and translation process. Differently, in *S. cerevisiae*, a remarkable number of terms are up-regulated during batch cultivation, as previously well established through microarray studies. In order to capture changes in the architecture of the transcriptional network, we used the Reporter Feature algorithm [26]. In Figure 4B the metabolites around which significant changes occur are shown, listed according to their pathway [26]. Interestingly, in *S. stipitis,* the reporter metabolites around which more transcriptional differences occur are involved in amino-acids metabolism, tRNA related processes and lipid-related metabolism. tRNA related process are over-represented in both yeasts during batch cultivations, probably as a result of the higher specific growth rate. Reporter metabolites involved in amino-acids metabolism are most represented during batch cultivations of *S. stipitis*, probably signifying its faster growth and increased biomass yield under this condition. Reporter metabolites involved in the dNTPs biosynthesis instead, appear to be more significant during chemostat conditions for *S. stipitis* and the same pattern is observed for most lipids. Interestingly, analysis of reporter metabolites of the central carbon metabolism indicated an increased significance of isocitrate, citrate and malate in *S. stipitis* during batch conditions; which is consistent with the high TCA activity observed under glucose excess conditions .

As our goal was to capture differences in transcripts of genes of the central carbon metabolism and provide a quantitative comparison of the transcriptome of the two yeasts, we described the data obtained through RNA-seq in a quantitative fashion using FPKM (fragments per kilobase per million sequenced reads). This parameter quantifies the expression level of a certain mRNA according to its abundance and normalized it by the number of reads per samples and the gene length [27].

**Figure 5:**
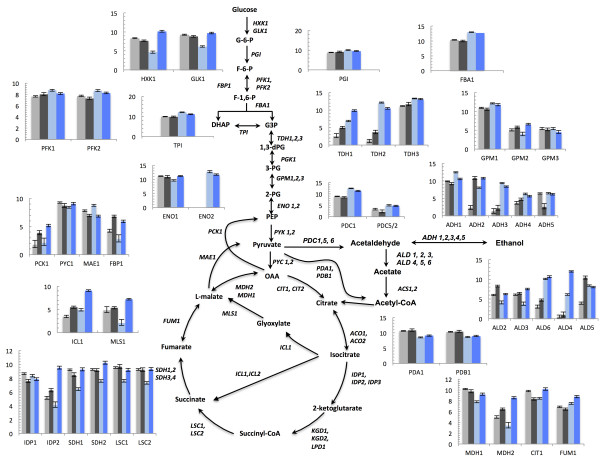
**mRNAs levels of genes involved in central carbon metabolism.** Expression level is based on FPKM and shown in the plot is the log_2_ FPKM. RNA-seq data were processed using the read-aligner Stampy and using Cuffdiff for statistical analysis. Legend: Light grey = *S. stipitis* batch; Dark grey = *S. stipitis* chemostat; Light blue = *S. cerevisiae* batch; Dark blue = *S. cerevisiae* chemostat.

We searched for genes whose expression changed in the two conditions between the two yeasts. Transcript levels of genes involved in central carbon metabolism are shown in Figure 5. Glycolytic genes show several differences in their expression patterns. In *S. cerevisiae*, the expression level of *HXK1* appears to be sensitively glucose repressed, as its mRNA in batch cultivations showed remarkable lower expression (half than in chemostat) and a similar behavior is observed for *GLK1*. In *S. stipitis*, where glucose repression is absent, there is almost no difference in mRNA levels of these genes under the two conditions. Other glycolytic genes that show to be sensitive to the presence of glucose in *S. cerevisiae* are *ENO1* and *TDH1*. Interestingly, in *S. stipitis*, *TDH1* and *TDH2* genes are less expressed than in *S. cerevisiae*. A lower expression is also reported for the genes *FBA1* and *TPI,* despite the difference is not as remarkable as that reported for *TDH1* and *TDH2*. Pentose phosphate pathway genes (data not shown) do not show significant changes, accordingly to what is known about the housekeeping function of *ZWF1*[28] and the constant levels of mRNA of the genes *TKL1* and *GND1*[29]. Different expression patterns between the two yeasts are found for genes of TCA and glyoxylate cycle. In *S. cerevisiae* it is possible to observe differences in mRNA levels between batch and chemostat cultivation due to the inhibitory presence of glucose; mRNA levels of TCA genes, besides *IDP1*, are higher during chemostat cultivations when glucose repression is relieved. Differently, in *S. stipitis,* the FPKM of these genes do not change remarkably. A similar trend is observed for the main glyoxylate genes *MLS1* and *ICL1*: their mRNA levels in *S. cerevisiae* are overexpressed during chemostat cultivations, whereas in *S. stipitis* this difference is not remarkable, however, in *S. stipitis,* expression of *MLS1* is higher than that reported by *S. cerevisiae* in batch. The gene *ACS1*, involved in acetyl-CoA metabolism, behaves in a similar fashion: in *S. cerevisiae* its mRNA is more abundant during chemostat cultivations while its expression does not change significantly in *S. stipitis;* additionally, mRNA of *ACS1* appears to be, similarly to *MLS1*, more abundant in *S. stipitis* during batch compared to *S. cerevisiae*. Genes of the PDH complex *PDA1* and *PDB1* are present in higher amount in *S. stipitis* than in *S. cerevisiae*. Expression of the PDC complex is also different as mRNAs corresponding to the *S. stipitis* genes *PDC1* and *PDC2* are not as much expressed as the corresponding *S. cerevisiae* genes. The mRNAs of genes involved in anaplerotic reactions show different patterns of expression between *S. cerevisiae* and *S. stipitis*. While *PCK1* and *PYC1* are increased in chemostat for *S. cerevisiae*, in *S. stipitis* it not possible to observe the same trend.

RNA-seq data provided information about the different levels of expression of genes of the central carbon metabolism between the two conditions, indicating that transcriptional regulation of central carbon metabolism differ, qualitatively and quantitatively, between the two yeasts.

### *In silico* analysis of transcription factors

**Figure 6:**
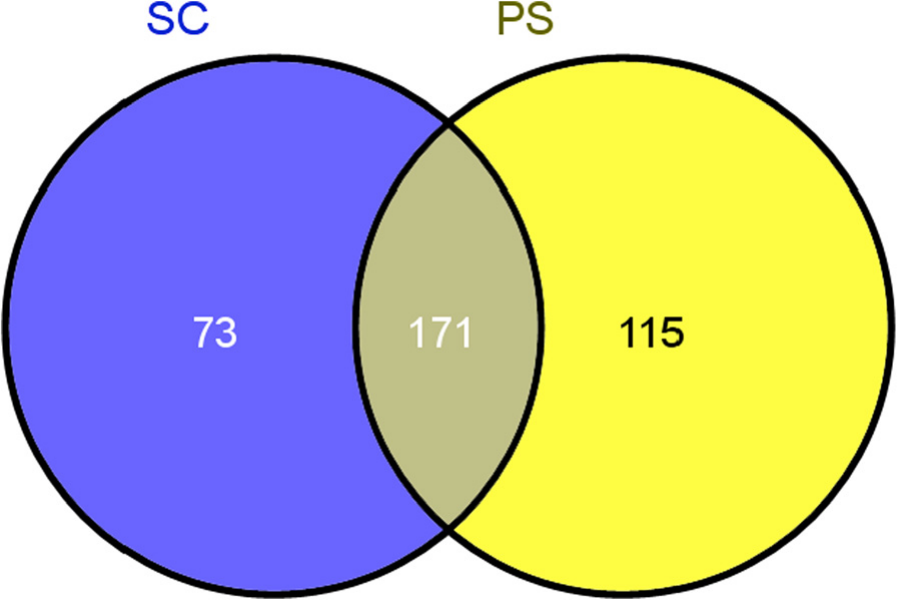
**Families of Transcription Factors shared between the two yeast species.** The available list of transcription factors was downloaded and, using the software OrthoMCL, orthologs families were reconstructed, leading to the identification of unique and shared families. *S. cerevisiae* = Sc; *S. stipitis* = Ps.

To gain insight into the regulation of the Crabtree negative yeast we performed a comparative *in-silico* analysis of Transcription Factors (TF) of the two yeasts. We used the Fungal Transcription Factor Database [30] to identify the known transcription factors (TF) of both yeasts. Here, we identified families of shared and unique transcription factors between *S. cerevisiae* and *S. stipitis* by performing clustering based on orthologs families [31] Figure 6 shows the Venn’s diagram of families of transcription factors (TFs): while 171 families are shared between the two yeasts, 73 families (most of them encompassing unique genes) are unique for *S. cerevisiae* and 115 for *S. stipitis*. The analysis of TFs found unique for *S. stipitis* uncovered that most of the TFs in these families are poorly characterized ORF. Most of them are putative or predicted proteins sharing conserved domains with *Debaromyces hansenii*, *Spathaspora passalidarum* or members of *Candida* spp., however, even in these yeasts, their function remains unknown. Many of the predicted transcription factors are Zn finger protein having GAL4-like domains. Some are involved in tRNA processing and maturation. Among the annotated ones we found a transcriptional activator of nitrogen-regulated genes, a beta-1,6-N-acetyl-glucosaminyl-transferase involved in transcriptional processes and the subunit A of HAP3.1 transcription factor which directly interacts with the more characterized regulator HAP2. Moreover transcription factors involved in arginine metabolism, lactose metabolism and fluconazole resistance (FRC1) have also been uniquely found in *S. stipitis*.

To attempt uncovering the reason at the origin of a different transcriptional regulation of genes involved in the central carbon metabolism, we looked at unique transcription factors of *S. cerevisiae* involved in central carbon metabolism that are reported in Table 6. The finding that INO2 and INO4, involved in regulation of phospholipid biosynthesis and amino acid biosynthesis [32,33], were found to be unique in *S. cerevisiae,* might suggest a different regulation of the phospholipid and fatty acids biosynthetic pathway. The ORF YOR344C, corresponding to the transcription factor SGC1 was identified as unique in *S. cerevisiae*; interestingly, this TF seems to function as a transcriptional activator in Ty1-mediated gene expression [34] and was identified as suppressor of defects caused by mutation in the glycolytic regulator gcr1 [35], showing that activity of SGC1 is required to have maximal enolase expression. The finding of a glycolytic regulator to be uniquely present in *S. cerevisiae* might be linked to the finding that RGT1, a TF involved in regulating expression of glucose transporters (HXT) during growth on glucose, is also unique for *S. cerevisiae*. Additionally, the protein MIG2 and MIG3 are also uniquely found in *S. cerevisiae*. Among specific TF of *S. cerevisiae* we also found GSM1, a poorly characterized transcriptional regulatory protein (YJL103C) probably involved in energy metabolism [36] and two gluconeogenic regulators: RMD5 and VID22. While RMD5 has a negative role in the gluconeogenic pathway [37,38], VID22 is involved in FBP1 transport and degradation. The other unique TFs found in *S. cerevisiae* are proteins involved in cell cycle progression, tRNA and mRNA maturation and processing, sulfite resistance, arginine and methionine metabolism, hypoxic genes regulators, nitrogen metabolism and filamentous growth. The finding that TFs involved in controlling the expression of glycolytic and gluconeogenic genes were found to be uniquely present in *S. cerevisiae* indicated that the two yeast show a different regulation exerted by glucose.

**Table 6:** **Transcription factors involved in central carbon metabolism uniquely identified for *S. cerevisiae***

***ORF***	***Name***	***Description***
**YDR123C**	*INO2*	Positive regulatory required for depression of the phospholipid biosynthetic enzymes, regulated by OPI1.
**YOL108C**	*INO4*	
**YOR344C**	*SGC1*	Serine-rich protein binding E-boxes of glycolytic genes and contributes to their activation. Has been found to suppress the *gcr1* requirement for enolase, glyceraldehyde-3-phosphate dehydrogenase, phosphoglycerate kinase, phosphoglycerate mutase, and pyruvate kinase gene expression. It is necessary for maximal enolase expression
**YKL038W**	*RGT1*	Transcription factor that regulates expression of several glucose transporter (HXT) genes in response to glucose.
**YGL209W**	*MIG2*	Regulatory protein, involved in glucose repression of the *SUC* genes.
**YER028C**	*MIG3*	Transcriptional repressor controlled by Snf1 involved in controlling the transcription of *SIR* genes. Also involved in the response to toxic agents.
**YLR373C**	*VID22*	Glycosylated integral membrane protein involved in fructose-1,6-bisphosphatase (FBPase) transport and degradation.
**YDR255C**	*RMD5*	Conserved protein involved in the degradation of the gluconeogenic enzyme fructose-1,6-bisphosphatase; also required for sporulation. Negative regulator of gluconeogenesis.
**YJL103C**	*GSM1*	Putative zinc cluster protein of unknown function; proposed to be involved in the regulation of energy metabolism.

## Conclusions

With the aim of describing the metabolism of *S. stipitis* during aerobic growth, we performed a systems-level comparison between the Crabtree negative yeast and *S. cerevisiae* during aerobic growth on glucose. To our knowledge, this is the first time that the metabolism of *S. stipits* is investigated in details during batch and chemostat cultivations on glucose. Despite the differences in genome evolution and metabolism of these two yeast species [39] we sought to identify patterns of similarity by applying a systemic approach based on high-throughput techniques. What clearly emerges from our study is the absence of a fermentative mode for *S. stipitis*. This behavior is very different from *S. cerevisiae* where is the amount of glucose to determine its metabolic mode (respiratory or fermentative). This is in agreement with what has previously been reported for *S. stipitis* concerning the onset of ethanol fermentation, which is dependent on oxygen availability [6]. The flux network of *S. stipitis* under both conditions show similarities to that observed for *S. cerevisiae* during purely respiratory growth, however, minor differences during batch and chemostat cultivations condition were identified [40]. The estimation of intracellular fluxes based on ^13^C-labeling uncovered differences through the anaplerotic reactions of pyruvate carboxylase and malic enzyme, showing lower values in *S. stipitis* compared to those showed by *S. cerevisiae* during oxidative growth. Interestingly, the flux through the oxidative part of the PPP is found to be higher during batch cultivations and higher than that reported in *S. cerevisiae* during oxidative growth. This might be explained with a higher biomass yield on substrate reported for *S. stipitis. S. stipitis* showed increased pyruvate import into the mitochondria, increased flux through the PDH and TCA reactions, indicating that the Crabtree negative yeast mainly fuel the TCA through the conversion of pyruvate to acetyl-CoA via the PDH reaction.

Through metabolome analysis it was possible to assess and quantify differences in the levels of intracellular metabolites of the two yeasts during batch cultivations. Total amino-acid levels are slightly lower in *S. stipitis* while adenine content is found to be higher. This result might be in agreement with the observed increase in biomass yield and higher specific growth rate. Carbohydrate metabolism shows several differences, mainly in the content of polyols. This might indicate that these compounds are used as storage carbohydrate or, as observed in *Aspergillus nidulans*, can play a role in maintaining the redox balance [18]. Relevant amounts of citramalate are found in *S. stipitis*, which is not present in *S. cerevisiae*. Differences in fatty acids and phospholipids are also found. The increased choline content could suggest a different regulation of the phospholipid metabolism as, through the analysis of unique transcription factors, we found the TFs INO2 and INO4 to be absent in *S. stipitis*. Analysis of RNA-seq data uncovered different transcriptional regulation of central carbon metabolism of the two yeasts, indicating that, in *S. stipitis*, transcriptional regulation is not dependent on glucose concentration. Most mRNAs of *S. stipitis* do not change their expression under the two conditions, in contrast to *S. cerevisiae* where, in the shift towards respiratory conditions, a significant number of genes change remarkably their expression [15]. The analysis of transcription factors was particularly relevant in identifying unique transcription factors of *S. cerevisiae* potentially involved in controlling the onset of fermentative metabolism (Rgt1 and Sgc1). Several families of transcription factors were found to be unique for *S. stipitis;* unfortunately, even though the *S. stipitis* genome was sequenced in 2007, most of these ORF have not been assigned a function yet.

Despite the non-direct relationship between mRNA levels, metabolites and fluxes, our attempt to integrate data coming from these different techniques uncovered a robust consistency in identifying a one-mode phenotype of *S. stipitis,* sharing similarities to the patterns found in *S. cerevisiae* during purely respiratory growth. However, through integrated system-level analysis, it was possible to identify differences between the main metabolic mode of *S. stipitis* and the oxidative growth of *S. cerevisiae*, to highlights non-obvious difference during batch cultivation conditions and identify potential transcription factors involved in controlling the different response to glucose excess.

## Methods

### Yeast strains

The *Scheffersomyces stipitis* strain used in this work was CBS 6054 obtained by T. Jeffries. The *Saccharomyces cerevisiae* strain CEN.PK113-7D was used as wild-type strain for comparison.

### Batch and chemostat cultivations conditions for physiological characterization

The cultivations have been conducted in DasGip fermentors (DasGip, Jülich, Germany) in batch and chemostat modes, for each mode triplicates cultures were grown. A mineral salt medium [41] was used. The medium is composed of (per liter): (NH_4_)_2_SO_4_, 5 g; KH_2_PO_4_, 3 g; MgSO_4_·7H_2_O, 0.5 g; Antifoam 289 (A-5551, Sigma–Aldrich, St. Louis, MO, USA), 0.05 mL; trace metals, 1 mL and vitamins, 1 mL trace metal solution. The trace metal solution consisted of (per liter): EDTA (sodium salt), 15 g; ZnSO_4_·7H_2_O, 0.45 g; MnCl_2_·2H_2_O, 1 g; CoCl_2_·6H_2_O, 0.3 g; CuSO_4_·5H_2_O, 0.3 g; Na_2_MoO_4_·2H_2_O, 0.4 g; CaCl_2_·2H_2_O, 0.45 g; FeSO_4_·7H_2_O, 0.3 g; H_3_BO_3_, 0.1 g and KI, 0.1 g. The pH of the trace metal solution was adjusted to 4.0 with 2 M NaOH prior to heat sterilization. The vitamin solution contained (per liter): biotin, 0.05 g; p-amino benzoic acid, 0.2 g; nicotinic acid, 1 g; Ca-pantothenate, 1 g; pyridoxine-HCl, 1 g; thiamine-HCl, 1 g and myo-inositol, 25 g. The pH of the vitamin solution was adjusted to 6.5 with 2 M NaOH. The vitamin solution was filter sterilized and stored at 4°C [41]. The medium was supplemented 20 gL^-1^ of Glucose as carbon source. The precultures were used to inoculate the fermentors to an initial OD_600_ of 0.05.

The batch cultures were performed in 1.0 L DasGip stirrer-pro® vessels with a working volume of 0.7 L. Agitation was maintained at 600 rpm using a magnetic stirrer integrated in the BioBlock®, which maintained the temperature at 30°C. The aeration was set to 1 vvm. The pH of the medium was maintained at 5.0 by automatic addition of 2 M KOH. The temperature, agitation, gassing, pH and composition of the offgas were monitored and controlled using the DasGip monitoring and control system. Dissolved oxygen was monitored with an autoclavable polarographic oxygen electrode (Mettler Toledo, Columbus, OH, USA). The effluent gas from the fermentation was analyzed for real-time determination of oxygen and CO_2_ concentration by DasGip fedbatch pro® gas analysis systems with the off gas analyzer GA4 based on zirconium dioxide and two-beam infrared sensor.

For the chemostat cultivations, the medium described above (except that Glucose was in a concentration of 10 gL^-1^) was fed with a constant dilution rate of 0.1 hr^-1^ and aeration was set to 1 vvm. The working volume was kept at 0.5 L by a peristaltic effluent pump. Samples were taken after a metabolic steady state (defined as constant values of CO_2_ and O_2_ in the off-gas, as well as constant biomass concentration for at least five residence time) was achieved.

### Batch and chemostat cultivations conditions for flux analysis upon ^13^C Glucose labeling

Metabolic flux distribution was analyzed after feeding the cultivations with 13C labeled glucose. The media used and the fermentation system was the same as described in paragraph 2.2 but glucose concentrations was 5 gL^-1^ for batch and 2 gL^-1^ for chemostat. Samples in the batch were taken in the mid-exponential phase; samples from chemostat cultivations were taken after a metabolic (see above) first and an isotopic steady state (at least 5 residence time after feeding with labeled glucose) were reached.

### ^13^C labeling experiments and flux data analysis

Flux distribution analysis was performed upon growth on ^13^C-labeled glucose during batch and chemostat cultivations. For batch cultures, the medium was added with 5 gL^-1^ of 100% D-Glucose-1-13C (^13^C > 99%; Isotec/ Sigma-Aldrich) and samples from triplicate cultures were withdrawn from mid-exponential phase (OD600nm = 2,5). For chemostat cultures, after reaching a metabolic steady state, the original medium containing 2gL^-1^ naturally labeled glucose was replaced by chemically identical medium, but where the glucose was replaced by 100% D-Glucose-1-^13^C (^13^C > 99%; Isotec/ Sigma-Aldrich).

The labeling pattern of proteinogenic amino acids was determined calculating the summed fractional labeling (SFL) as described in Gombert *et al.*[14]. This calculations are based on the model previously described [42].

### Analytical methods

Cell dry weight was measured by filtering a known volume of the culture through a pre-dried and pre-weighed 0.45 μm pore size nitrocellulose filter (Supor®-450 Membrane Filters, PALL Life Sciences, Ann Arbor, MI, USA). The filters with the biomass were washed with water, dried for 15 min in a microwave oven at 150 W and weighed again. The optical density was determined at 600 nm using a Genesys 20 Spectrophotometer, Thermo Scientific, Wilmington, DE, USA).

The concentrations of glycerol, ethanol, acetate, succinate and pyruvate were analyzed by an isocratic HPLC (UltiMate® 3000 Nano/Capillary Autosamplers, Dionex, Sunnyvale, CA, USA) with an Aminex HPX-87 H ion exchange column (Bio-Rad, Hercules, USA) at 65°C using 5 mM H_2_SO_4_ as mobile phase at a flow rate of 0.6 mL min^-1^. Glucose, glycerol and ethanol were measured with a refraction index detector whereas succinate, acetate and pyruvate with an ultraviolet–visible light absorbance detector.

### Treatment of sample for metabolites analysis: quenching and extraction

Sample from each condition were withdrawn from mid-exponential phase (OD600nm 2,5) and added to a 75% v/v cold methanol buffered with 12.5 mM Tricine pH 7.4, kept at −40°C in an Ethanol Bath. Pellet were harvested by centrifugation 4 min at 10,000 g at −20°C and froze on liquid nitrogen. The quenched samples were sent to Metabolon (Metabolon, Inc., Durham, NC) for extraction based on methanol/chloroform and analysis through GC-MS and LC-MS-MS. Metabolome profiling was performed as previously described [43,44]. The untargeted metabolic profiling platform employed for this analysis was based on a combination of three independent platforms: ultrahigh performance liquid chromatography/tandem mass spectrometry (UHPLC/MS/MS) optimized for basic species, UHPLC/MS/MS optimized for acidic species, and gas chromatography/mass spectrometry (GC/MS). Metabolites were extracted from the samples using a methanol-based solvent, dried and reconstituted in chromatography solvent. The reconstituted extracts were divided into three portions and resolved using the three chromatography platform systems coupled to mass spectrometry. Samples were derivatized using trimethylsilane prior to injection into the GC/MS instrument. Metabolites were identified by matching ions chromatographic retention index and mass spectral fragmentation signatures with reference library entries created from standard metabolites. For ions that were not covered by the standards, additional library entries were added based on their unique retention time and ion signatures.

### Total RNA extraction and RNA sequencing

For RNA sequencing, a 25 mL sample was withdrawn from the fermentor in the mid-exponential phase (OD_600_ = 2.5). The sample was cooled immediately in ice and centrifuged at 2°C and 5000 rpm for 5 min, the supernatant discarded and the biomass immediately frozen in liquid nitrogen. The total RNA was extracted from cells through mechanical disruption with glass beads, digested with DNAse and purified using the RNeasy kit (Qiagen, Hilden, Germany). The quality of the RNA was assayed using a BioAnalyzer (Agilent Technologies, Palo Alto, CA, USA). 10 ng of total RNA were sent to Lab for Life for RNA sequencing based on the Illumina Solexa platform. For construction of the library the Illumina TrueSeq kit was used.

### Illumina sequencing data processing

The pair-end reads were firstly checked their quality using SolexaQA [45]. The reads that heave Phred score less than 20 and were trimmed out using BWA trimming algorithm [46]. The trimmed high quality reads that have length less than 25 bases were filtered out. The pre-processed reads were aligned on the reference genome of *S. stipitis* CBS6045 and *S. cerevisiae S288c* using Bowtie-Tophat software v1.3.3. With this method, more than 90% of the pre-processed reads could be aligned on their reference genome [47,48]. The RNA-seq raw data of *S. stipitis* were deposited at NCBI SRA database with accession number SRS308058. For RNA-seq raw data of *S. cerevisiae* were retrieved from our study [49].

### FPKM calculation, gene ontology and reporter metabolites analysis

We used the Cuffink software to estimate the gene expression level based on the parameter FPKM (fragments per kilobase of exon per million fragments mapped) and Cuffdiff to determine differential gene expression [50,51]. The adjusted p-values from differential gene expression analysis were further overlaid on the Gene Ontology networkand genome-scale metabolic model of iIN800 [52] and iSS884 for integrated analysis. The reporter algorithm [26] was applied to evaluate the significant features (GOterms, metabolite) in the different conditions. The results were selected based on reporter p-value cutoff 0.01 for GOterms and 0.05 for Metabolites.

### Orthologs group and TFs analysis

The protein sequences based translated from the genome sequence of *S. stipitis* CBS6045 and *S. serevisiae* S288c were used to identify orthologous group through OrthoMCL software version 1.4 using default parameters [31]. The result from the orthologous clustering can be found in Additional file 1. The collection of transcription factor of the both genome was retrieved from the Fungal Transcription Factor Database [53], and, based on the orthologs families reconstructed, a comparative of analysis of TF of the two yeasts was performed.

## Competing interests

The authors declare no competing financial interests.

## Authors’ contributions

MP, JN and IN designed the experiments, MP performed the experiments, MP and IN analyzed the data, IN, MP, MU and JN discussed and interpreted the data. All authors read and approved the final manuscript.

## Supplementary Material

Additional file 1 The orthologous family analysis result from OrthoMCL software. Click here for file
